# Enhancing Indoor Inertial Pedestrian Navigation Using a Shoe-Worn Marker

**DOI:** 10.3390/s130809836

**Published:** 2013-08-02

**Authors:** Mitja Placer, Stanislav Kovačič

**Affiliations:** 1 Harpha Sea, Čevljarska Ulica 8, Koper 6000, Slovenia; 2 Fakulteta za Elektrotehniko, Univerza v Ljubljani, Tržaška 25, Ljubljana 1000, Slovenia; E-Mail: stanislav.kovacic@fe.uni-lj.si

**Keywords:** indoor positioning, strapdown inertial navigation, pedestrian dead reckoning, marker tracking, unscented Kalman filter, unit quaternion space

## Abstract

We propose a novel hybrid inertial sensors-based indoor pedestrian dead reckoning system, aided by computer vision-derived position measurements. In contrast to prior vision-based or vision-aided solutions, where environmental markers were used—either deployed in known positions or extracted directly from it—we use a shoe-fixed marker, which serves as positional reference to an opposite shoe-mounted camera during foot swing, making our system self-contained. Position measurements can be therefore more reliably fed to a complementary unscented Kalman filter, enhancing the accuracy of the estimated travelled path for 78%, compared to using solely zero velocities as pseudo-measurements.

## 1. Introduction

Indoor pedestrian positioning, a prominent example of where Global *Navigation Satellite System* (GNSS) solutions come up short in terms of performance [1], is a fast growing segment with great potential. This kind of positioning could prove itself to be as useful for the general public (e.g., context-aware applications in airports, shopping malls, libraries, museums, subways, *etc.*) as for professional users (e.g., helping firefighters and first responders to navigate in low visibility conditions).

Various indoor pedestrian positioning methods have been proposed through the years. Most of the approaches rely either on a modified environment (e.g., radio beacons or fixed visual markers), or some a priori knowledge about it (e.g., radio fingerprinting). In emergency, life-critical scenarios, where time and reliability are of utmost importance, such solutions could be easily stretched beyond their limits. Beauregard [2] has listed a number of demanding, for many technologies prohibitive technical requirements in these worst case scenarios, and also pointed out the time-consuming deployment and calibration of UWB beacon-based positioning systems. Poor visibility and unfavorable lighting conditions can also render unreliable other substantially different approaches, such as a fiducials-free computer vision-based SLAM positioning system—possibly resulting even in completely false position estimation [3]. Rantakokko *et al.* [4] observed that a robust and accurate first responder positioning system for urban operations requires the use of a multi-sensor approach.

## 2. Related Work

With the help of modern self-contained inertial sensors the aforementioned shortcomings could be overcome to a certain degree at least. However, the hard to ignore issue of low cost Inertial Measurement Unit (IMU)-based personal navigation systems lays in the inaccuracy of their microelectromechanical system (MEMS)-type sensors. Even with theoretically perfect initial alignment, accurate position tracking can only be successfully performed for a few seconds using commercial grade inertial sensing alone [5], due to cubic-in-time positional error growth caused by angle rates and accelerations integration inherent to the Strapdown Inertial Navigation System (SDINS) algorithm.

To limit the error growth characteristics of low-cost IMU-based pedestrian Inertial Navigation System (INS), also known as Pedestrian Dead Reckoning (PDR), an inherent property of the human gait has been widely exploited—the fact that cyclically one foot at a time stays still on the ground for a short period of time (stance phase) while the opposite one is moving (swing phase) [6]. By taking advantage of this property positional error growth can be decoupled from time, making it linearly proportional to the number of steps taken. Many approaches have been tried in this direction to date, differing by algorithms used, sensors choice and their placement. Foxlin's [7] NavShoe concept represents a substantial upgrade to foot-mounted IMU-based PDRs by introducing the addition of zero velocity updates (ZUPT) as pseudo-measurements to an extended Kalman filter (EKF) during stance phase. Introducing ZUPTs as measurements into the EKF, instead of simply resetting velocity in the SDINS algorithm to zero, brings the substantial advantage of a retroactive correction of the state vector. More recently, the ZUPT approach was used by Alvarez *et al.* [8] in a waist-worn inertial personal navigation system that can be precise enough for some applications.

**Figure 1.**
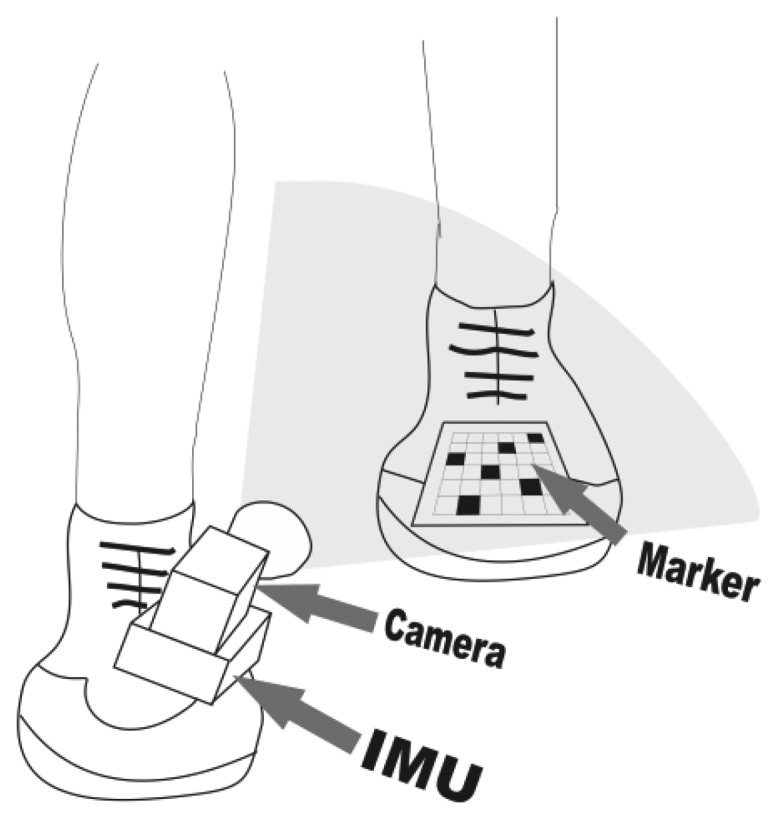
Our proof-of-concept PDR.

Considerable research has been carried out recently in the hybrid indoor positioning field. Having in mind that most of the presented hybrid approaches are not self-contained or rely on environmental features [9] and that a lot of research has been done in heading estimation improvement for PDR, we sought to engage an innovative way to somehow enhance the original ZUPT approach, especially in difficult, typical first-responder scenarios. The result is our low-cost, proof-of-concept hybrid PDR depicted in Figure 1. Computer vision was chosen as the aiding modality because of its complementarity to inertial sensing as Corke *et al.* pointed out in [10], while the desire to simultaneously decrease the dependence of the system on environmental features led us to think in the direction of a wearable marker. To the best of our knowledge, no general pedestrian navigation platform, that would allow a seamless blending of visual sensory information, is available at the moment. The modular multi-sensor pedestrian location and navigation platform with integrated data timestamping that Morrison *et al.* describe in [11] seems very close to this aim, but it does not support video input in its present iteration.

Our approach is similar to the one proposed by Do and Suh with their Gait Analysis System [12] for the fact that it uses a shoe-mounted IMU with a rigidly connected camera. The crucial difference comes in the placement of the visual marker. We opted for a shoe-fixed marker on the opposite foot, serving as a positional reference to the IMU-camera-compass (IMUCC) unit, mounted onto the other shoe. Using a self-attached visual marker as a positional reference for a combined IMUCC unit is a novel approach, since visual markers are usually pre-deployed into the environment.

We therefore propose a novel, self-contained machine vision-aided hybrid PDR aiming to improve foot trajectory estimation in an IMU-based PDR system with ZUPTs. The idea is to minimize foot trajectory error during the swing phase, particularly during slow or disturbed walking, when the swing phase, and thus the error integration time, may last longer. We achieve our goal by taking advantage of a novel visual marker-based setup, where traditional environmental markers are being replaced by a user-worn marker, fixed on the user's shoe, while an IMUCC unit is placed on the opposite one. From the time it enters in the camera's field of view, the marker's pose with respect to the camera can be determined by means of an augmented reality (AR) machine vision algorithm and from then on it can serve as a positional reference to the IMU, since there is a fixed, known spatial relationship between the camera coordinate frame and the IMU coordinate frame. Position measurements can be therefore fed to a complementary unscented Kalman filter (UKF), operating in a unit quaternion space in feedback configuration.

## 3. System Description

**Figure 2.**
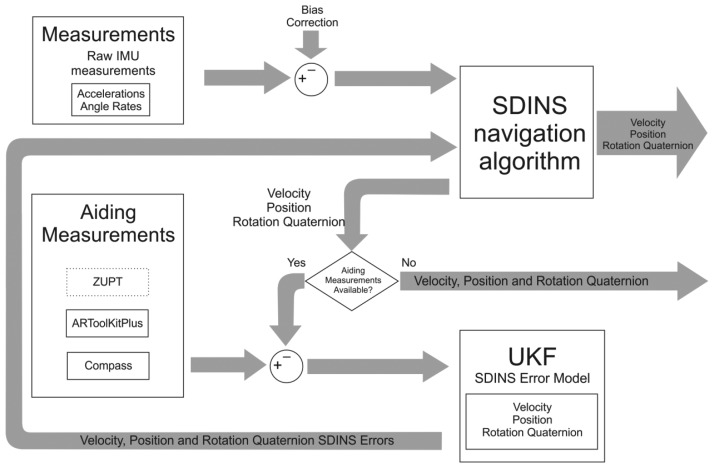
Flowchart of the proposed algorithm.

In this section a description of the proposed algorithm will be given. Its schematic representation is shown in Figure 2. Emphasis will be put on aiding measurements and filtering algorithms, whereas the SDINS navigation algorithm in quaternion approach will be treated in the Appendix section at the end of this article.

### 3.1. Coordinate Systems and Notation

We make use of the following Cartesian, orthogonal coordinate reference systems (frames) throughout the article:

- *Inertial (i-frame)* is the inertial frame, fixed with respect to the stars with the origin in the center of the Earth. All inertial measurements are done with respect to this frame.
- *Earth (e-frame)* is the Earth centered and fixed frame. This frame is of limited use in our case, but of great importance using higher accuracy IMUs in long distance and high dynamics outdoor missions.
- *Local NED (n-frame)* is the local level frame at the SDINS computed position, following the NED (north-east-down) notation.
- *Navigation (nav-frame)* is the local level frame at the first SDINS computed position. In our case, using a low-cost IMU, not capable of Earth-rate sensing, in low dynamics conditions for short periods of time, we neglect Earth's curvature and rotation, making possible to assume the alignment of the nav-frame and n-frame. We use the NED notation for this frame. Camera pose is being calculated with respect to this frame.
- *IMU (b-frame)* is the coordinate frame of the body—in our case the frame of the IMU, which is attached to the shoe. All the inertial measurements are being measured in this frame.
- *Camera (cam-frame)* is the coordinate system of the camera, which is rigidly connected to the IMU unit. The z axis is pointing along the optical axis, starting from the optical center of the camera, its x axis perpendicular to the z axis to the right.
- *Reference Marker (mref-frame)* is the coordinate system of the first recognized marker during the swing phase, which serves as a reference to the subsequent ARToolKitPlus measurements that occur later during the current swing phase.
- *Marker (m-frame)* is the marker coordinate frame. It is fixed onto the user's opposite shoe with the z axis pointing out from the marker and the x axis pointing perpendicular to the z axis to the left.
- *Platform (p-frame)* is the SDINS software frame in which the transformed inertial quantities (accelerations and angular rates) are being solved. In the ideal case the p-frame and n-frame would be parallel, but because of the errors, inherent to inertial sensors, a discrepancy arise. The p-frame axes configuration copies that of the n-frame.

All frames are right-handed, the third axes are thus defined with the first two. In this article superscript is used to denote the coordinate system in which a variable is represented. Bold text is used for vector and matrix variables.

### 3.2. Visual Position Estimation Using a Shoe-Mounted Marker

The main idea behind our approach was motivated by the fact that SDINS positional uncertainty grows cubically over time. Developing a method to somehow anchor subsequent foot position measurements during swing phase, when SDINS calculations keep losing accuracy, to a prior point in time, when accuracy was greater, was our goal. The use of a shoe-worn marker would allow us to perform positional measurements with accuracy that is substantially decoupled from time, similarly to using an outdoor operating GNSS receiver. The cubical position error growth of the inertial-only SDINS solution during the swing phase should therefore become limited to the sum of the positional error, which occurred up to the time of the reference marker image acquisition and the inherent ARToolKitPlus [13] positional measurement error. Using visual position estimation, an enhanced navigation solution is expected, when longer SDINS integration times occur (*i.e.*, during slow walk), compared to a SDINS algorithm using only ZUPT pseudo-measurements.

**Figure 3.**
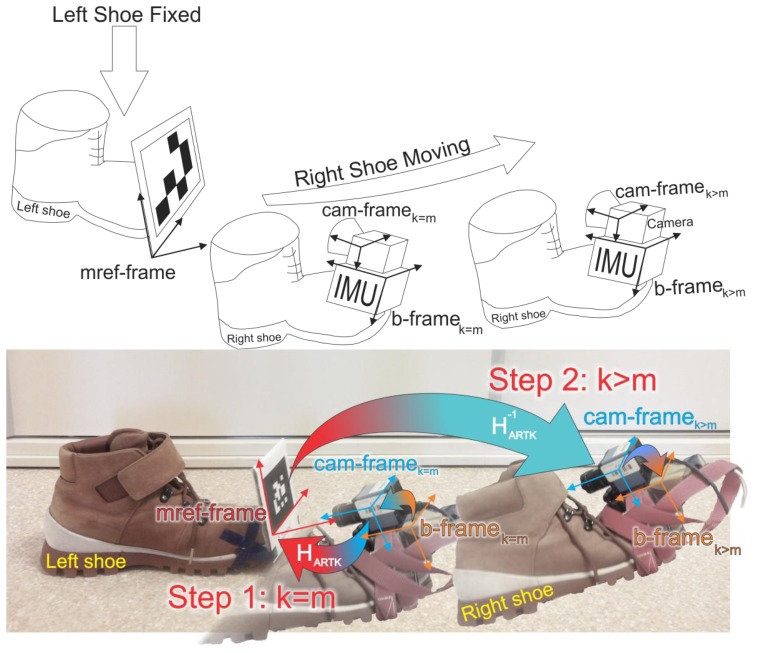
Schematics of our two-step pose calculation process, yielding the IMU position in the navigation frame as the final result. If *m* denotes the moment when the marker is first seen during the current swing phase, then mref-frame is being defined at time step *k = m*, while successive b-frames are being calculated at time steps *k > m*, when the marker is being detected in the acquired video frames.

The coordinate systems transformations involved, leading to the actual positional measurement being fed to the UKF, can be thought of as a two-step process (Figure 3):

1. When the marker is first recognized in an image during the swing phase, its 3D pose is calculated with the aid of the IMU frame calculated pose in the nav-frame, the known IMU-Camera lever arm and the current ARToolKitPlus measurement of the pose of the marker in the cam-frame. We call this resulting frame the reference marker coordinate frame (mref-frame).
2. From now on the system's computational workflow reverses with respect to the first step. The inverse of the ARToolkitPlus homogeneous matrix is used to perform the transform from the previously calculated mref-frame, expressed in the nav-frame, to the cam-frame and again the known IMU-Camera lever arm transformations are used at the end for calculating the actual homogeneous matrix, describing the pose of the IMU frame in the navigation frame.

The visual marker-based relative 3D positional measurement architecture that we are using relies on the well-known ARToolKitPlus AR framework, which is based on the pose estimation algorithm developed by Schweighofer and Pinz [14]. Before an image can be efficiently used as a measurement, the camera unit has to be calibrated to compensate for optical distortions caused by the lens. With the aid of the Camera Calibration Toolbox for MATLAB [15] and a printed checkerboard pattern we have determined the intrinsic parameters of the camera-lens combination and rectified the images before they were fed to the ARToolKitPlus for image processing.

A common characteristic of optical tracking AR systems with fiducial markers are their viewing angle W-shaped rotation error functions [16,17]. With this in mind we tried to mount the marker in a way that it would remain outside of lower accuracy regions throughout the whole swing phase, because not only position, but the whole pose information (*i.e.*, including orientation) was used later in calculations. When fixing the marker onto the shoe, we therefore rotated the marker for a few degrees towards the camera around its yaw axis and slightly increased its pitch, compared to a marker facing directly into the walking direction.

Our camera is rigidly connected to the IMU. Using the calibration algorithm developed by Hol *et al.* [18], high quality estimates of the relative orientation between the camera frame and the IMU frame could be determined. However, we chose to follow a more straightforward, yet effective approach: to a first approximation, in our paper we assume that the camera axes are either completely parallel or perpendicular to the IMU axes. Translation offsets between the center of the IMU and the sensor of the camera were accurately measured with the aid of a Vernier caliper. Knowing both rotational and translational relationships between the b-frame and cam-frame, homogeneous matrix transforms needed to convert back and forth between the two coordinate systems can be determined.

### 3.3. Data Synchronization

Having three separate measurement data streams of mutually dependent quantities, one for visual marker pose measurements, one for inertial measurements and one for magnetic compass heading measurements, gives rise to the data synchronization issue. The most accurate and straightforward solution in our case would be to use a hardware-based time synchronization mechanism for the three streams, similarly to [18]. Not having such a system at hand, we opted for a more Ad-Hoc approach since all computations are made off-line. We logged inertial serial data sentences at 156 Hz, video frames were taken at a fixed rate of 15 Hz and magnetic compass readings at 20 Hz. While recording all data streams (inertial, video and compass) simultaneously we completed a quick rotation of the combined IMUCC unit, making sure the marker does not leave the camera's field of view during the move. We then applied our SDINS algorithm to the recorded inertial data, extracted the computed IMU orientations and transformed them into camera Roll-Pitch-Yaw (RPY) orientations. Since visual marker trackers give the most accurate rotation estimation results for roll rotation (around the z axis of the cam-frame) [17], we had accomplished the time-synchronization procedure with regard to this rotation. For synchronizing the compass data stream we took advantage of the roll readings in the compass output sentences. At this point we had three different data streams representing the same quantity (IMUCC unit roll), one lagging another for an unknown amount of time. We then had to resample the data sets to the same frequency—we chose to resample the less-frequently sampled camera orientation and compass roll data streams to the same frequency as the IMU data to prevent precision reduction of the final synchronization result. We used quadratic instead of linear interpolation to achieve a slightly smoother resampled curve. The final step of the data synchronization procedure was to determine the lag among data streams by comparing the derivatives of the orientation curves extracted from all three modalities.

### 3.4. Filtering Algorithms

#### 3.4.1. The Error State Vector

Complementary filtering involves system error estimation through system error modeling. Therefore, the states used in our complementary filter architecture are SDINS velocity and position errors in the n-frame and quaternion attitude error between the p-frame and n-frame. All states used in a Kalman filter are considered to be white-noise driven signals. Any time-correlated driving noise should be properly shaped. In most cases, a first-order Gauss-Markov process model is accurate enough for modeling such errors. The state vector is then augmented with additional states to accommodate these additional colored noise states. To use this approach is particularly important in long lasting missions using sensors with non-negligible sensor drift, where the filter is augmented with accelerometer and gyro bias states. However, because the noise power spectral density (PSD) curve of the IMU outputs showed negligible drifts for the duration of our proof-of-concept experiments, we considered accelerometer and gyro biases as zero mean white noises in our article for simplicity and clarity reasons.

Therefore, considering velocity, position and attitude as the main quantities of interest in a navigation solution and sensor biases as time uncorrelated states, we can define the following error state vector for our complementary UKF:

(1) $$x={\left[\Delta v_{x}^{n},\Delta v_{y}^{n},\Delta v_{z}^{n},\Delta r_{x}^{nav},\Delta r_{y}^{nav},\Delta r_{z}^{nav},q_{0},q_{1},q_{2},q_{3}\right]}^{T}$$

where $\Delta v^{n}={\left[\Delta v_{x}^{n},\Delta v_{y}^{n},\Delta v_{z}^{n}\right]}^{T}$ is SDINS velocity error in the n-frame, $\Delta r^{nav}={\left[\Delta r_{x}^{nav},\Delta r_{y}^{nav},\Delta r_{z}^{nav}\right]}^{T}$ is the SDINS position error in the nav-frame and $q_{p}^{n}={\left[q_{0},q_{1},q_{2},q_{3}\right]}^{T}$ is a unit quaternion, representing the error rotation between the platform and navigation frame, which means its norm is of size one $\|q_{p}^{n}\|\equiv \sqrt{q_{0}^{2}+q_{1}^{2}+q_{2}^{2}+q_{3}^{2}}$, ${ℍ}_{1}\equiv \{{\text{q}}_{p}^{n}\in ℍ|\left\|{\text{q}}_{p}^{n}\right\|=1\}$.

As it can be observed above, the filter error state vector is composed of a translational part (Δ***v****^n^* and Δ***v****^nav^*) and a rotational part ( ${\text{q}}_{p}^{n}$). Representing the rotational part of the filter with quaternions means that common vector space UKF cannot be used for propagating the whole state vector in time, because of the unit quaternion departure from the unit sphere due to the addition and multiplication operation in the weighted mean procedure. We therefore chose to combine two separate versions of unscented transform (UT) operating on a common state vector for error state propagation, a translational UT in vector space for velocity and position error propagation, and a rotational UT in unit quaternion space for attitude error states propagation.

#### 3.4.2. Translational UKF in Vector Space

The unscented Kalman filter constitutes an alternative to the extended Kalman filter, which is a suboptimal implementation of the recursive Bayesian estimation framework applied to Gaussian random variables [19]. Developed for nonlinear process and measurement models in estimation and control problems, it is based on the principle that it is easier to approximate a Gaussian distribution than it is to approximate an arbitrary nonlinear function, making cumbersome Jacobian or Hessian calculations, which are the base for derivative-based filters like it is the EKF, superfluous. In the UKF, sample points (also called sigma points) are propagated through a nonlinear system, but, unlike in particle filters, a minimal set of sample points is deterministically chosen to capture the posterior mean and covariance of a random variable up to the 2nd order.

A complementary filter operates on the navigation errors in the error state space, recursively estimating them, making it possible to correct navigation states in the total state space SDINS. We have not employed any small angle assumption in the development of the algorithm. A complementary UKF in a unit quaternion space was developed for the rotational (unit quaternion) part of the state vector propagation, while a vector space complementary UKF was used for the Euclidean vector space part of the state vector. Hereafter in this chapter we will describe the fundamental algorithm of the latter.

Considering the following discrete-time process governed by the nonlinear stochastic difference equation:

(2) $$x_{k}=f({\text{x}}_{k-1},k)+w_{k-1}$$

with measurements ***z****_k_*:

(3) $$z_{k}=h({\text{x}}_{k},k)+v_{k}$$

x_k_ ∈ ℜ^n × 1^ being the state vector, **Z**_k_ ∈ ℜ^m×1^ the measurement vector, **f** the nonlinear system dynamic model, **h** the observation model, **w_k_** and **v**_k_ the process and measurement zero mean Gaussian noises with covariances given by **Q**_k−1_ and **R**_k_, respectively, an UKF tries to estimate the state vector **x**_k_ by the following procedure: given an n × n covariance matrix **P_k_** a set of 2n + 1 sigma vectors **χ**_i,k_ can be generated and column-wise concatenated to form the matrix **χ**_k−1_ of size n × (2n + 1):

(4) $$\chi_{k-1}=\lfloor \begin{array}{lll} {\hat{x}}_{k-1} & {\hat{x}}_{k-1}+{\left(\gamma \sqrt{P_{k-1}+Q_{k-1}}\right)}_{i|i=1,\ldots ,n} & {\hat{x}}_{k-1}-{\left(\gamma \sqrt{P_{k-1}+Q_{k-1}}\right)}_{i-n|i=n+1,\ldots ,2n} \end{array}\rfloor$$

where x̂_k-1_ is the distribution mean at time step k − 1 and γ is a composite scaling parameter. From Equation (4) one can observe that process noise **Q**_k_ is added to**P_k_** before the sigma points (vectors) are projected ahead in time. Sigma vectors **χ**_i,k−1_ are then propagated through the nonlinear function **f** to get the posterior sigma point vectors:

(5) $$\chi_{i,k}=f(\chi_{i,k-1},k),(i=1,\ldots ,2n+1)$$

where ***χ****_i,k_* denotes the *i*-th column of ***χ****_k_*. Applying a weighted sample mean and covariance of the ***χ****_i,k_* vectors, we get the predicted state vector ${\hat{\text{x}}}_{k}^{-}$ and its associated predicted covariance $P_{k}^{-}$:

(6) $${\hat{x}}_{k}^{-}=\overset{2n}{\underset{i=0}{\text{∑}}}W_{i}^{\left(m\right)}\chi_{i,k}$$

(7) $$P_{k}^{-}=\overset{2n}{\underset{i=0}{\text{∑}}}W_{i}^{\left(c\right)}\left\{\chi_{i,k}-{\hat{x}}_{k}^{-}\right\}{\left\{\chi_{i,k}-{\hat{x}}_{k}^{-}\right\}}^{T}$$

where *n* is the number of sigma points and *W_i_* are the corresponding weights given by the equations developed in [20]. By propagating the ***χ****_i,k_* sigma vectors through the measurement model ***h***:

(8) $$\gamma_{i,k}=h(\chi_{i,k},k),(i=1,\ldots ,2n+1)$$

we get the *i*-th column ***γ****_i_*_,_*_k_* of the matrix ***γ****_k_*. The predicted observation vector ${\hat{y}}_{k}^{-}$ and its predicted output covariance $P_{k}^{yy}$ are determined by applying weighted sample mean and weighted covariance computation as above for ${\hat{x}}_{K}^{-}$ and $P_{k}^{-}$ respectively.

#### 3.4.3. Rotational UKF in a Unit Quaternion Space

In contrast to vector quantities, rotations lie on a nonlinear manifold and quaternions, used in our system to represent them, are constrained to a unit radius hypersphere in a four-dimensional Euclidean space (a 3-sphere). This is the reason why quaternions are not closed for addition and scalar multiplication (operations that constitute the core of the weighted sum calculations in an UKF) and consequently why using unscented filtering directly with a unit quaternion attitude parametrization generally yields a non-unit quaternion estimate [21].

**Figure 4.**
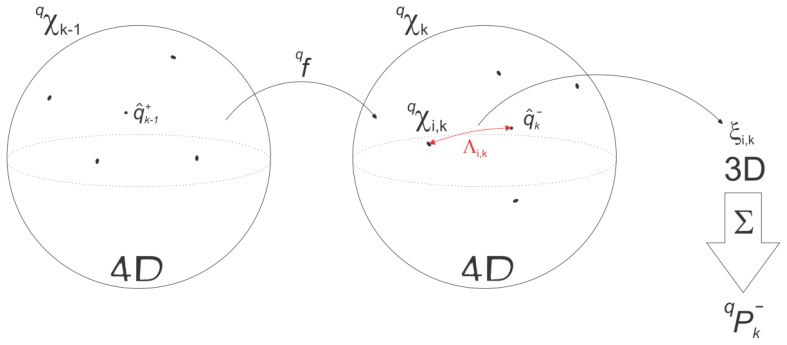
Schematic diagram of the rotational part of the UKF filter.

The original vector space UKF algorithm has thus to be modified accordingly to ensure that during the weighted sum of the unscented transform, the quaternion does not depart from the unit sphere. This was achieved with the help of the rotation vector attitude representation for sigma point rotation vector generation, followed by a quaternion-based weighted mean computation based on the quaternion distance metric formulated in [22] (Figure 4). A more in-depth description of the rotational filter algorithm is given below.

We chose to treat the quaternion noise $\delta {\hat{q}}_{k-1}^{+}$(the equivalent to ***w****_k_*_−1_ in the vector space UKF, where the superscript + denotes the post-update process noise estimate when gyro bias error is being considered) as a rotation vector, because this way the transformed sigma points are more narrowly scattered around the current estimate compared to the alternative noise representation as a vector part of the quaternion [23]:

(9) $$\delta {\hat{q}}_{i,k-1}^{+}=\left[\begin{array}{ll} \cos\left(\left|\xi_{i,k-1}\right|/2\right) & \xi_{i,k-1}\sin\left(\left|\xi_{i,k-1}\right|/2\right)/\left|\xi_{i,k-1}\right| \end{array}\right]$$

where $\xi_{i,k-1}=\gamma \sqrt{P_{k-1}+Q_{k-1}}$ denotes the i-th column three-component noise vector and $\delta {\hat{q}}_{i,k-1}^{+}$ is the resulting error quaternion. As can be seen we choose to apply process noise (with covariance ***Q****_k_*_−1_) before the process model. To avoid using addition and multiplication in the quaternion unit sphere domain, we use quaternion multiplication in sigma point generation instead, multiplying the quaternion error by the current quaternion estimate ${\hat{q}}_{k-1}^{+}$:

(10) χqi,k−1=δq^i,k−1+⊗q^k−1+

where *^q^****χ****_i,k_*_-1_ is the resulting transformed *i*-th quaternion sigma point. Using both the quaternion error and the quaternion error inverse to construct the set of sigma point quaternions, we ensure an evenly distributed set of points, lying on the unit sphere around the current quaternion estimate:

(11) χqk−1=⌊q^k−1+δq^i,k−1+⊗q^i,k−1+(δq^i,k−1+)−1⊗q^i,k−1+⌋

where $\chi_{k-1}^{q}$ denotes the set of sigma points for the rotational part of the error state vector.

Dealing with a combined translation-rotational UKF, we would like to emphasize that (2*n* + 1) sigma points have to be generated for the combined filter, meaning that using the rotation vector representation for rotational sigma point generation, 19 sigma points have to be generated (*n* = 9). This set of transformed quaternions is then propagated forward in time through the rotational part of the process model *^q^****f*** yielding the new set *^q^****χ****_k_*:

(12) χqi,k=fq(χqi,k−1,k)

where *i* denotes the *i*-th column of the respective sigma point set. No additional noise is being considered in the equation above, since process noise is embedded into the sigma points already and is thus represented in the sigma points distribution.

The predicted mean quaternion part ${\hat{q}}_{k}^{-}$ of the error state vector ( x^qk−) is then determined as the barycentric mean with renormalization:

(13) x^qk−=∑i=02nWqiχi,k|∑i=02nWqiχi,k|

The associated predicted rotational covariance Pqk− is computed by first finding the distance ***Λ***_i_ between the single sigma quaternion and the predicted mean quaternion:

(14) Λi,k=χqi,k⊗(x^qk−)−1

Each quaternion distance ***Λ****_i_*_,_*_k_* is then converted to the equivalent distance rotation vector ***ξ**_i,k_* with the following equation:

(15) $$\xi_{i,k}=\frac{\Lambda_{i,k}^{'}\left|\xi_{i,k}\right|}{\sin\left(\frac{\left|\xi_{i,k}\right|}{2}\right)}$$

where $\Lambda_{i,k}^{'}$ denotes the three component imaginary part of the quaternion distance ***Λ****_i_*_,_*_k_* and the distance rotation vector norm |*ξ_i,k_*| is given by:

(16) $$\left|\xi_{i,k}\right|=2\arccos(\Lambda_{i,k}^{0})$$

where $\Lambda_{i,k}^{0}$ denotes the real part of the quaternion distance ***Λ****_i_*_,_*_k_*. Finally, the predicted covariance $P_{k}^{-}$ is calculated with:

(17) Pqk−=∑i=02nWiξi,k(ξi,k)T

Propagating the quaternion sigma points through the measurement model:

(18) γqi,k=h(χqi,k,k),(i=1,…,2n+1

and applying weighted sample mean and weighted covariance computation, the predicted quaternion observation ${\hat{y}}_{k}^{q-}$ and predicted output covariance $P_{k}^{qyy}$ are obtained, respectively.

#### 3.4.4. Measurement Modes

Since we are using three different measurement modalities, namely ZUPT, visual position measurement (ARTK) and heading measurement (Figure 2), our system has to deal with three distinct measurement modes, each with a different observation matrix ***H***. In the ZUPT pseudo-measurement mode (we make use of the “pseudo” prefix throughout the article because these observations are not actual measurements, but the assumed zero velocities) the observation matrix selects the velocity states, in the ARTK position measurement the ***H*** matrix selects the three position states, while in the case of a heading measurement the four orientation quaternion states are selected from the filter error state vector, which are consequently converted to a rotation vector during the UKF sigma points calculations. This back and forth conversion between the nine and ten states is required because of the covariance computation within the rotational part of the filter [24].

The measurement switching module sets the ARTK measurement mode each time the marker is recognized in the image and a position measurement is therefore available, while an effective and straightforward gyro signals thresholding technique similar to Foxlin's approach in [7] is used to enable the ZUPT pseudo-measurement mode. Foxlin's one-heading-measurement-per-step approach was used for heading measurement update—we performed it once per step to limit the effects of the colored environmental magnetic noise, at the time of the first ZUPT pseudo-measurement in the detected stance phase, because at that time the compass measurement should have been stabilized already. In the measurement update stage of the filter ZUPT mode was set to higher priority than the ARTK mode, because of the lower uncertainty of its pseudo-measurements, while the time update-only stage was performed during the swing phase without any external observations, to update the states error covariance in the UKF due to inertial sensor errors.

Dealing with complementary filter architecture, we have to stress the fact that the measurements ***z***_k_ involved in the update stage of the filter are actually difference quantities *δ****z****_k_*, *i.e.*, errors between the measurements and the respective estimated total states. Since linear velocities belong to the Euclidean vector space, arithmetic subtraction can be performed to get the actual measurement *δ^v^_Z__k_* fed to the filter when performing in the ZUPT pseudo-measurement mode:

(19) δvzk=zZUPTk−HZUPTx^k(SDINS)−

where *^ZUPT^****z****_k_* is the zero velocity pseudo-measurement at time step *k*, *^ZUPT^****H****_k_* is the ZUPT observation matrix and ${\hat{x}}_{k(\text{SDINS})}^{-}$ is the predicted total state vector of the SDINS algorithm at time step *k*, which is the SDINS total state vector, corrected by the values calculated in the time update stage of the complementary UKF. With zero pseudo-measurements *^ZUPT^****z****_k_* the equation above can be rewritten as:

(20) δvzk=−HZUPTx^k(SDINS)−

In heading measurement mode we are dealing with rotation measurements represented by unit quaternions, which are not mathematically closed for subtraction. We thus opted for the following quaternion multiplication approach to get the heading error measurement to be fed to the UKF in a complementary configuration. Heading was first extracted from the SDINS attitude quaternion $q_{b}^{p}$ by converting it to the rotation matrix notation, then subtracted from the compass heading measurement and the result converted to a difference rotation quaternion δ^q^***z***_k_,which represents the measured difference rotation quaternion to be fed to the UKF:

(21) δqzk=(HHeadingx^q−k(SDINS))⊗zHeading−1k

where *^Heading^****z****_k_* is the combined measurement attitude, composed by the predicted SDINS pitch and roll and the compass corrected heading measurement, *^Heading^****H****_k_* is the heading observation matrix and *δ^q^****z****_k_* is the rotational heading error between the current estimated heading and the heading, observed by the magnetic compass.

It has to be noted that heading measurements are presented as attitude measurements to the UKF in the heading measurement mode, therefore the generally nonlinear measurement transfer model ***h*** is represented by a linear matrix transform *^Heading^****H***, which directly mirrors the measurements to the respective quaternion attitude states. Consequently, distance vectors ***ξ**_i,k_* belonging to the quaternion sigma point set *^q^****χ****_i,k_*, belong to the propagated set *^q^****γ****_i,k_* as well. We take advantage of this property in the cross correlation matrix calculations below.

In the ARTK measurement mode the difference *δ^r^****z***_k_ between the IMU's ARTK measured position and the SDINS estimated position of the IMU sensor is being fed to the UKF:

(22) δrzk=zARTKk−HARTKx^k(SDINS)−

where *^ARTK^****z****_k_* is the positional measurement at time step *k*, *^ARTK^****H****_k_* is the ARTK observation matrix and ${\hat{x}}_{K(\text{SDINS})}^{-}$ is the same as described for the ZUPT measurement mode above.

**Figure 5.**
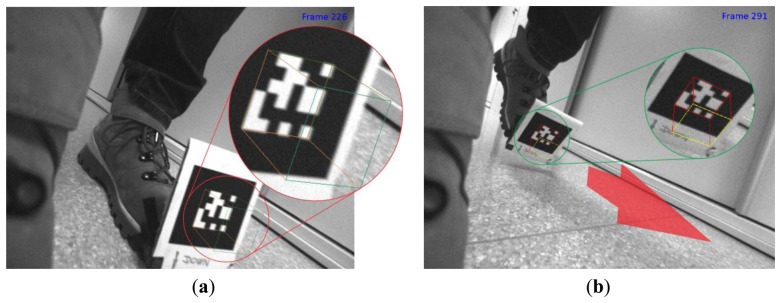
Typical rejected ARTooKitPlus measurements. (**a**) 3D cube overlay of a low confidence ARTooKitPlus marker pose estimation—notice the imperfect 3D cube overlay on the right edge of the marker. (**b**) A typical moving marker image during stance phase of the IMUCC-equipped foot with an otherwise perfectly determined marker pose.

Regarding the ARTK measurement mode, a few words need to be devoted to its triggering. First of all, only pose measurements with high confidence are taken into account in the UKF, since frames (measurements) with lower ARToolkitPlus confidence tended to produce poor 3D cube overlays (Figure 5a), which meant 3D marker pose estimations were inaccurately determined for some reason. Since low confidence ARToolKitPlus measurements occurred rarely, simply discarding those measurements proved to be an effective strategy to cope with this phenomenon. Secondly, ARTK measurement mode has to be triggered only when the marker-equipped foot is stationary on the floor to allow precise 3D pose measurement of the reference marker coordinate frame and also of all successive camera coordinate frames during the swing phase of the IMUCC-equipped foot. However, marker pose measurements are often available during the stance phase, just before heading and subsequent ZUPT measurement mode triggering occur (Figure 5b). To effectively reject these inadequate measurements, velocity thresholding was implemented, *i.e.*, if the calculated velocity of the IMUCC unit fell below a certain value, then the ARTK triggering signal was not passed through to enable the ARTK measurement mode in the UKF. To allow ARTK positional measurements during midair interrupted swings, when the foot would almost hover over the floor, some other kind of triggering strategy has to be employed, e.g., SDINS height triggering or using a shoe with an integrated ground contact sole switch.

#### 3.4.5. Kalman Gain and Measurement Update Equations

The UKF gain ***K****_k_* is obtained with:

(23) $$K_{k}=P_{k}^{xy}{\left(P_{k}^{inn}\right)}^{-1}$$

where $P_{k}^{inn}$, given the measurement (observation) noise covariance ***R***, is the innovation covariance $P_{k}^{inn}$:

(24) $$P_{k}^{inn}=P_{k}^{yy}+R$$

and $P_{k}^{xy}$ is the cross correlation matrix, calculated as weighted cross correlation between the posterior sigma point vectors ***χ****_i_*_,_*_k_* and the predicted observation sigma vectors ***γ****_i_*_,_*_k_*:

(25) $$P_{k}^{xy}=\overset{2n}{\underset{i=0}{\text{∑}}}W_{i}^{\left(c\right)}\left\{\chi_{i,k}-{\hat{x}}_{k}^{-}\right\}{\left\{\gamma_{i,k}-{\hat{y}}_{k}^{-}\right\}}^{T}$$

The cross correlation matrix $P_{k}^{qxy}$ for the rotational part of the filter cannot be calculated using quaternion subtraction. Hence, the rotation vector is used as the distance measure again:

(26) $$P_{k}^{qxy}=\sum_{i=0}^{2n}W_{i}^{\left(c\right)}\xi_{i,k}{\xi_{i,k}}^{T}$$

We can define the error state vector $\Delta {\hat{x}}_{k}^{n+}$ and covariance $P_{k}^{+}$ as:

(27) $$\Delta {\hat{x}}_{k}^{n+}=K_{k}(z_{k}-{\hat{y}}_{k}^{-})$$

(28) $$P_{k}^{+}=P_{k}^{-}-K_{k}P_{k}^{inn}K_{k}^{T}$$

where ***z****_k_* is the new observation at time step *k*.In the ARTK measurement mode the error measurement quaternion and the predicted error measurement quaternion are first converted to the rotation vector notation, before being subtracted in ( $z_{k}-{\hat{y}}_{k}^{-}$). Thereafter, the last three states of the error state vector $\Delta {\hat{\text{x}}}_{k}^{n+}$ have to be reconverted to the quaternion notation, resulting in the error state $\Delta {\hat{x}}_{k}^{n+}$ composed of the upper vector part $\Delta^{v}{\hat{x}}_{k}^{n+}$ (velocity and position error states) and the lower rotational part x^qn+k.

Since we are dealing with a complementary filter architecture, SDINS states have to be updated by the error state vector $\Delta {\hat{x}}_{k}^{+}$. We refresh the SDINS states after a measurement update stage occurs, as described below, leaving the error states of the complementary filter in a zero error state:

(29) $$\Delta v^{n}={\left[0,0,0\right]}^{T},\Delta r^{n}={\left[0,0,0\right]}^{T},q_{p}^{n}={\left[1,0,0,0\right]}^{T}$$

We accomplish the update for the predicted vector part x^vk(SDINS)− of the SDINS state vector with:

(30) x^vk(SDINS)n+=x^vk(SDINS)n−+Δx^vkn+

while the predicted rotational part x^qk(SDINS)− of the SDINS state vector is being updated by the measurement with:

(31) x^qk(SDINS)+=x^qk+⊗x^qk(SDINS)−

#### 4. Experimental Results

The following hardware is used in our proof-of-concept PDR system:

- low-cost Analog Devices ADIS 16354AMLZ IMU
- 3-axis tilt-compensated magnetic compass Ocean Server OS-5000
- grayscale video camera The Imaging Source DMK 41 AF02 with a Computar 3.5–8 mm 1:1.4 1/3″ CS lens

All data preprocessing and computations are performed offline with main algorithms running in MATLAB Simulink environment. Experiments were conducted to evaluate the methods we proposed. The intrinsic parameters of the camera-lens combination and the turn-on biases of the inertial sensors were determined as described in Sections 3.1 and 3.2, respectively. MATLAB Simulink was used to perform offline computations.

Accelerometer and gyro triads error covariance matrices (cov(∇) and cov(ε), respectively) were determined by logging several minutes of IMU data, while leaving the IMUCC unit at rest:

(32) $$\mathrm{cov}(\nabla )=10^{-3}.\left[\begin{array}{ccc} 0.3586 & 0.0050 & 0.0301\\ 0.0050 & 0.4013 & -0.0617\\ 0.0301 & -0.0617 & 0.4442 \end{array}\right]\mathrm{cov}(ɛ)=\left[\begin{array}{ccc} 0.0054 & -0.0004 & 0.0002\\ 0.0004 & 0.0051 & 0.0002\\ 0.0002 & 0.0002 & 0.0051 \end{array}\right]$$

Since visual marker pose estimation accuracy is proportional to the marker physical size, we opted for a relatively big (79.3 mm wide), but still feasible marker, for our proof-of-concept PDR system.

The following parameters were used in our experimental setup:

(33) $$\begin{array}{l} {\text{R}}_{\text{ZUPT}}=0.01\times I\\ {\text{R}}_{\text{Heading}}=0.1\times I\\ {\text{R}}_{\text{ARTK}}=0.05\times I \end{array}$$

where ***R****_ZUPT_*, ***R****_Heading_* and ***R****_ARTK_* denote the ZUPT, heading and ARTK measurement noises, respectively. These specific values were chosen to reflect the actual accuracy of the singular attribute, while avoiding possible numerical instabilities.

### 4.1. Preliminary Experiment with Marker Fixed on the Floor

**Figure 6.**
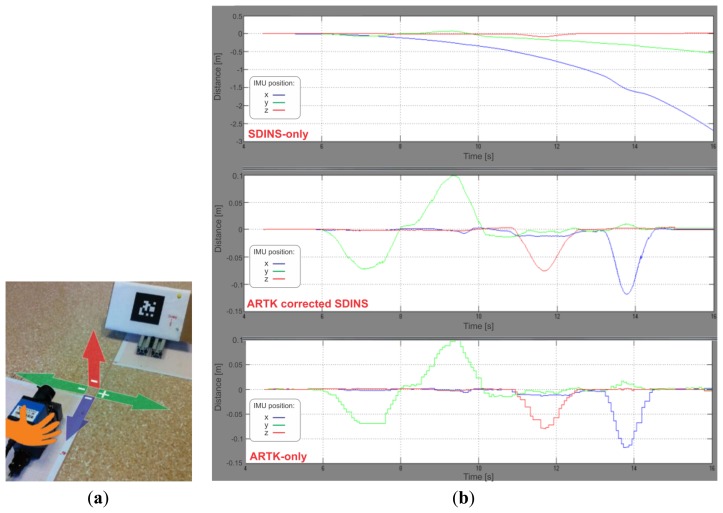
(**a**) Our first experimental setup. The IMU-Camera unit facing the fixed marker on the floor with arrows showing the directions of the movements completed, which coincide with the navigation frame axes in this case. The colors of the arrows and the imprinted signs are consistent with the curves, plotted on the graphs on the right. (**b**) Simulation results of the preliminary experiment.

Not having a 3D position measurement device at hand, we first decided to check the behaviour of our system by executing a round trip sequence (*i.e.*, always returning to the starting point) of movements—left, right, up and backward—with the IMU-camera unit in hand, while having the marker in a fixed, tilted position on the floor (Figure 6a). The expected result was a smooth (due to the high rate SDINS calculations) and drift-free (due to the drift-free visual measurements) positional trajectory. We focus here on the positional part of the navigation solution since user position is of greater importance than velocity and orientation in a pedestrian navigation system. Figure 6b shows the results of this 11 seconds-lasting experiment. The upper graph depicts the results of the SDINS-only solution, the graph in the middle shows the final result, obtained with our UKF visual sensor fusion technique, while the graph at the bottom represents the positional ARToolKitPlus measurements expressed in the reference navigation frame with the IMU-camera offsets taken into account. As it can be observed from the graphs, x position coordinate (yellow curve) drifted for almost 3 m, while y position coordinate (violet) drifted for approximately 0.5 m in the SDINS-only graph due to inaccurate gravity vector subtraction—compared to the other two graphs, which are drift-free.

### 4.2. Slow Walking Experiment

Having checked that the system improves the SDINS-only solution when being used for prolonged periods, we wanted to try it in a real slow walking scenario, to assess the amount of positional error correction during slow paced pedestrian navigation. This second experiment was performed walking slowly down the corridor of our lab, travelling with the IMUCC-equipped foot for 14.09 m in 44 s.

After having preprocessed all the data, ZUPT triggering parameters were set by trial and error:

(34) $$\begin{array}{c} \Delta \omega_{x}=\Delta \omega_{y}=\Delta \omega_{z}=\pm 8{}^{\circ}/s\\ T_{\text{ZUPT}}=47\;\text{samples} \end{array}$$

where Δ*ω_x_*, Δ*ω_v_* and Δ*ω_z_* are angular rate thresholds and *T_ZUPT_* is the time threshold after which the ZUPT mode is turned on, if all three angular rates remain under their respective thresholds. We did not need to use acceleration data to accomplish effective ZUPT mode triggering.

**Figure 7.**
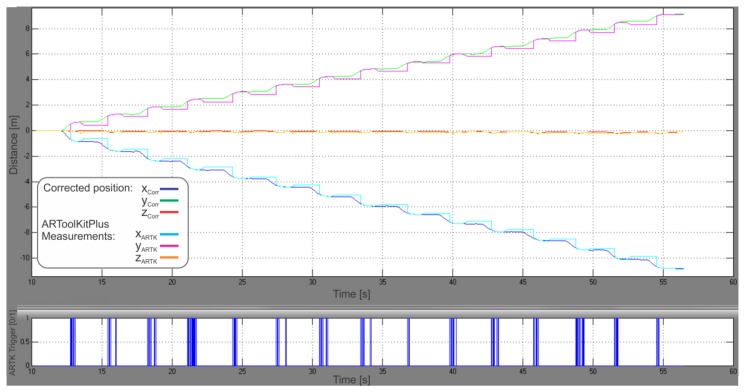
ARToolKitPlus-corrected positional navigation solution. The edgier red, green and dark blue curves are ARToolKitPlus measurements being fed to the UKF at the moments, represented by the blue spikes in the graph beneath.

**Figure 8.**
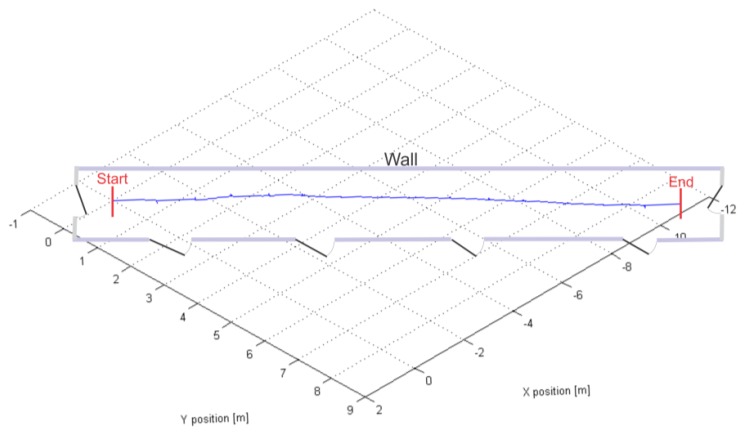
2D top view graph of the reconstructed experimental slow walk. The path started at the entrance to our Lab and following the wall finished at the other end of the corridor.

Figure 7 shows the positional graph of our hybrid PDR system with ARTK measurement mode enabled, calculated for the center of the IMU frame in the navigation frame. Fifteen steps done with the IMUCC-equipped foot can be recognized in the upper graph. The x and y position coordinates have a 90° clockwise rotated “V”-letter shape due to the IMUCC significant initial yaw in our experiment, imposed by the desired facing direction of the camera. The z coordinates are negative due to the NED convention employed for the nav-frame. ARTK position measurements were fed to the UKF at the moments, represented by blue spikes in the graph below in Figure 7. The final calculated point in 3D space is 14.15 m distant from the starting point, resulting in a 6 cm (0.43%) travelled distance error. Figure 8 shows the reconstructed experimental slow walk in a 2D top view representation.

**Figure 9.**
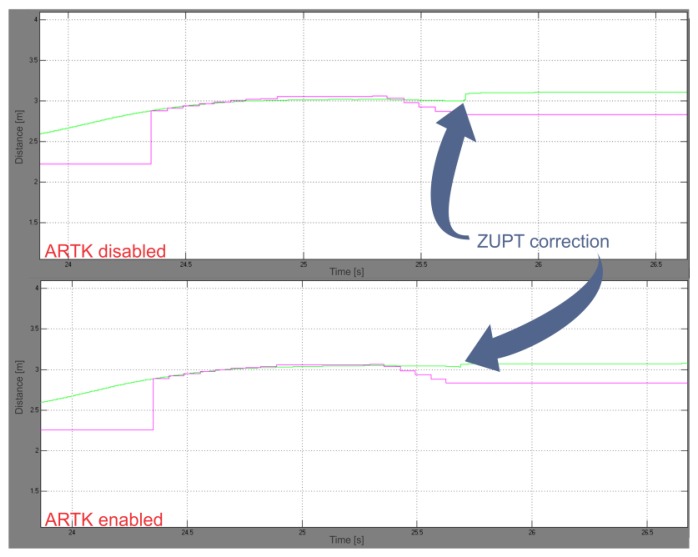
Close-ups of the two positional graphs obtained with the slow walking experimental data with ARTK mode disabled (**upper graph**) and ARTK mode enabled (**lower graph**). Sensible reduction in ZUPT-induced position correction is indicated by the arrows.

We repeated the calculations on the earlier acquired experimental data with ARTK measurement mode disabled, without altering all the remaining parameters, to be able to compare our proposed solution to a state-of-the-art PDR. A comparison of both positional graphs showed a reduction in ZUPT position corrections in the ARTK-enabled graph (Figure 9).

**Figure 10.**
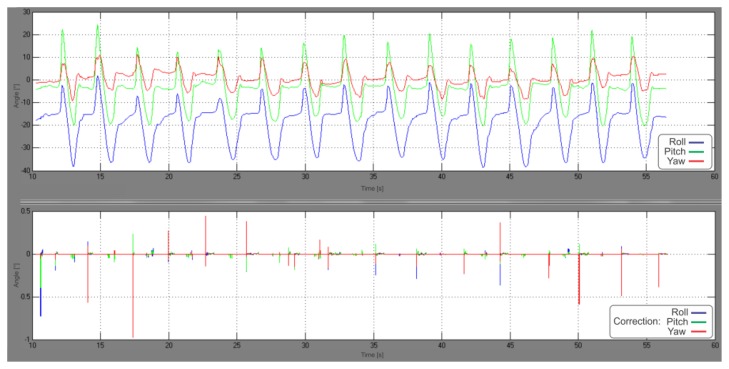
IMU frame orientation during the slow walking experiment, converted to Euler angles (**above**). Rotational corrections, made by the complementary UKF, converted to Euler angles (**below**).

The upper graph in Figure 10 shows the IMU-frame orientation during the slow walking experiment, while the graph below in Figure 10 depicts the rotational corrections, made by the rotational part of the UKF. Velocity error values, observed for each step at the beginning of the stance phase, just before ZUPT triggering occur, are presented in Table 1. Significant improvement in velocity norm error reduction (25% by comparing the means) is evident from Table 2, where corresponding statistical data is shown.

The final calculated point in 3D space for the ARTK-disabled SDINS system, therefore aided by ZUPT and compass measurements only, is 14.36 m distant from the starting point, resulting in a 27 cm (1.92%) travelled distance error. Comparing it to the 6 cm error with ARTK mode enabled, an 78% increase in accuracy can be observed in our slow walking experiment with our proposed hybrid PDR system.

## 5. Discussion

A significant improvement (25%) in velocity estimation has been observed during the experiments through beginning-of-stance-phase data analysis (Figure 9), where smaller ZUPT position corrections were observed in the ARTK-enabled graph, which meant greater velocity vector accuracy was achieved during vision-aided navigation. The improvement in velocity vector estimation is the result of a more efficient gravity vector subtraction, *i.e.*, better IMU-frame orientation estimation made possible by ARTK visual position measurements through the correlations developed in the UKF error states covariance matrix. The operational correctness of the rotational part of the filter can be observed also through the last two graphs on Figure 6b, where a great improvement in curve smoothness can be noted in the UKF graph compared to the sole ARToolKitPlus measurements, due to the IMU high sample rate and proper orientation states correction performed by the rotational part of the UKF.

We would like to stress the fact that navigation accuracy improvement is expected to be inversely proportional to the pace of the user and that we have chosen to test our system in a slow pace walk because it would represent a plausible, real-use scenario in which a substantial improvement in navigation accuracy would arise. Conversely, in a normal or fast pace walking, less or even no improvement is expected, due to the shorter SDINS integration time during swing phase. Moreover, using the same visual setup, image blurring would also occur due to the higher camera travel speed, making marker pose estimation less accurate. The mentioned effects of walking speed on accuracy improvement could therefore constitute the subject of further experimental investigation.

Besides walking speed, there is another limitation of our system—the fact that our visual aiding algorithm is based on the assumption of a stationary marker during stance phase of the marker-equipped foot. In fact, any movement of the marker would compromise the visual pose measurement of the marker and consequently the calculated pose of the camera. By examining the video acquired during our experimental walking, we could confirm that we succeeded in achieving no visible marker motion during the experiments in our controlled laboratory environment. However, if intended to be brought to practical use, the system should incorporate some kind of marker movement detection or limitation at least.

## 6. Conclusions

A proof-of-concept hybrid, inertial sensors-based indoor PDR system, aided by a novel position measurement technique, relying on a shoe-attached marker, has been proposed in this paper. Using a visual marker in this novel way was expected to enhance the navigation solution of an inertial-only PDR, while retaining independence from environmental markers or features, making the proposed approach potentially interesting, especially for first responders' tasks. However, additional sensors could be added to our system in cases when other sensory modalities would be available.

Since a comparison to other indoor pedestrian navigation systems, relying on external aids (e.g., environmental markers, radio fingerprinting, *etc.*) or environmental assumptions (straight walls, flat floor, ramps, *etc.*) would not be fair, we compared our system to an inertial PDR aided by ZUPT pseudo-measurements only, where an 78% reduction in error of calculated travelled distance has been shown during an indoor slow walking scenario in the experimental section, while a positioning improvement of 2.7 m was achieved in the axis with major positional error after just 11 s during the preliminary experiment using a marker placed on the floor. All the improvements have been achieved despite no modifications to the environment were made, no a priori knowledge about the environment was presupposed and with the only assumption being that about the marker being stationary during the stance phase of the marker-equipped foot.

Being proof-of-concept, our proposed hybrid PDR cannot be directly used in practice. However, in this paper we have shown that if brought to real-time operation and to a practically acceptable size and weight, the use of a hybrid PDR, aided by visual positional measurements, relying on a shoe-worn marker, can represent a viable solution for improving indoor PDR navigation for first responders in the first place.

## Figures and Tables

**Table 1.** IMU velocity vector norm error just before ZUPT triggering occurred, for each step of the slow walking experiment.

**Step #**	**Velocity Vector Norm Error (m/s) with ARTK Disabled**	**Velocity Vector Norm Error (m/s) with ARTK Enabled**
1	0.1144	0.0294
2	0.0872	0.0892
3	0.0980	0.0608
4	0.0325	0.0769
5	0.1293	0.0642
6	0.0736	0.0283
7	0.0533	0.0442
8	0.0713	0.0623
9	0.0914	0.0747
10	0.0793	0.0670
11	0.1030	0.0826
12	0.0463	0.0195
13	0.0617	0.0725
14	0.0294	0.0270
15	0.0604	0.0533

**Table 2.** Mean value, standard deviation and maximum value of data, presented in Table 1.

	**ARTK Disabled**	**ARTK Enabled**
Mean of velocity vector norm error (m/s)	0.0754	0.0568
Standard deviation of velocity vector norm error (m/s)	0.0291	0.0222
Minimum value of velocity vector norm error (m/s)	0.0294	0.0195
Maximum value of velocity vector norm error (m/s)	0.1293	0.0892

## Appendix: SDINS in Quaternion Approach

The SDINS is the main navigation algorithm in our system, based on IMU outputs. The SDINS algorithm takes IMU-measured accelerations (specific forces) ***a****^b^*=[*a_x_*, *a_y_*, *a_z_*] and angular rates $\omega_{rb}^{b}=\left[\omega_{x,}\omega_{y},\omega_{z}\right]$ in the b-frame with respect to the *i*-frame and outputs the navigation data (velocity ***v****^p^*, position ***r****^p^* and attitude $q_{b}^{p}(t)=\left[q_{0}(t),q_{1}(t),q_{2}(t),q_{3}(t)\right]$) in the p-frame, given the initial velocity, position and attitude. For attitude representation different parametrization options exist—Euler angles, DCM (Direction Cosine Matrix), modified Rodrigues parameters, quaternions and rotation vectors. We have chosen the quaternion approach since it is stated to be computationally effective and avoids singularity problems [25]. We define the SDINS equations in continuous total state space as:

A.1 $$\left[\begin{array}{c} {\dot{v}}^{P}\\ {\dot{r}}^{n\text{a}v}\\ {\dot{q}}_{b}^{p} \end{array}\right]=\left[\begin{array}{c} C\text{(}q_{b}^{p}\text{)}a^{b}+g^{p}\\ v^{p}\\ \frac{1}{2}\Omega_{b}^{n}\times q_{b}^{p} \end{array}\right]$$

where ***g****^p^* is the known gravitational acceleration vector in the platform frame, $C(q_{b}^{p})$ is the transformation matrix defined as:

(A.2) $$C(q_{b}^{p})=\left[\begin{array}{lll} {2q_{0}}^{2}-1+{2q_{1}}^{2} & 2q_{1}q_{2}+2q_{0}q_{3} & 2q_{1}q_{3}-2q_{0}q_{2}\\ 2q_{1}q_{2}-2q_{0}q_{3} & {2q_{0}}^{2}-1+{2q_{2}}^{2} & 2q_{3}q_{2}+2q_{0}q_{1}\\ 2q_{1}q_{3}+2q_{0}q_{2} & 2q_{3}q_{2}-2q_{0}q_{1} & {2q_{0}}^{2}-1+{2q_{3}}^{2} \end{array}\right]$$

and $\Omega_{b}^{n}$ the angular rate matrix:

(A.3) $$\Omega_{b}^{n}=\left[\begin{array}{cccc} 0 & -\omega_{x} & -\omega_{y} & -\omega_{z}\\ \omega_{x} & 0 & \omega_{z} & -\omega_{y}\\ \omega_{y} & -\omega_{z} & 0 & \omega_{x}\\ \omega_{z} & \omega_{y} & -\omega_{x} & 0 \end{array}\right]$$

In Equation (A.1) we intentionally ignored the Coriolis and Earth curvature effect part $-(2\omega_{ie}^{n}+\omega_{en}^{n})\times v^{n}$ in velocity computation since we use a consumer grade IMU in low dynamics conditions over limited distances making these effects drop below the IMU noise level.

Before using the IMU ***a****^b^* and $\omega_{rb}^{b}$ measurements, raw accelerometer and gyro data have to undergo some preprocessing to assess the turn-on biases. We used mean raw accelerometer data, collected during a stationary stage to calculate roll and pitch and perform leveling—determining the direction cosine matrix ***DCM****^init^*, describing the initial IMU attitude with respect to the navigation frame through gravity acceleration vector rotation:

(A.4) $${\text{DCM}}^{\text{init}}=\left[\begin{array}{ccc} \cos\theta & 0 & -\sin\theta \\ \sin\varphi \sin\theta & \cos\varphi & \sin\varphi \cos\theta \\ \cos\varphi \sin\theta & -\sin\varphi & \cos\varphi \cos\theta \end{array}\right]$$

where *θ* denotes the pitch (around *y* axis rotation) and *φ* the roll (around *x* axis rotation) of the stationary initial IMU pose. Knowing that the accelerometer mean output *F̂**^b^* represents bias corrupted true acceleration *F^b^*:

(A.5) $${\hat{F}}^{b}=F^{b}+\nabla^{b}(0)$$

we can calculate the bias vector of the accelerometers ∇*^b^*(0) by subtracting the known true acceleration (the gravity vector ***g***, expressed in the b-frame with the help of the ***DCM****^init^*) from the mean accelerometers outputs:

(A.6) $$\nabla^{b}(0)={\hat{F}}^{b}-{\text{DCM}}^{\text{init}}g$$

Generally, Earth rate is used to derive gyros' turn-on biases, but using a low-cost category IMU this term can be ignored and applying simple mean removal leads to turn-on bias-free data, which can be directly used for navigation.
